# Simulation data for an estimation of the maximum theoretical value and confidence interval for the correlation coefficient

**DOI:** 10.1016/j.dib.2017.07.045

**Published:** 2017-07-23

**Authors:** Paolo Rocco, Francesco Cilurzo, Paola Minghetti, Giulio Vistoli, Alessandro Pedretti

**Affiliations:** Department of Pharmaceutical Sciences, Università degli Studi di Milano, via G. Colombo, 71, I-20133 Milan, Italy

**Keywords:** Numerical simulation, Fisher transform, Skin permeability, Maximum theoretical correlation coefficient, Confidence interval around correlation coeffcient

## Abstract

The data presented in this article are related to the article titled "Molecular Dynamics as a tool for in silico screening of skin permeability" (Rocco et al., 2017) [1]. Knowledge of the confidence interval and maximum theoretical value of the correlation coefficient *r* can prove useful to estimate the reliability of developed predictive models, in particular when there is great variability in compiled experimental datasets. In this Data in Brief article, data from purposely designed numerical simulations are presented to show how much the maximum *r* value is worsened by increasing the data uncertainty. The corresponding confidence interval of *r* is determined by using the Fisher *r*→*Z* transform.

## Specifications Table

**Table:** 

Subject area	*Chemistry*
More specific subject area	*Chemometrics*
Type of data	*Table*
How data was acquired	*Numerical simulation*
Data format	*Raw, Analysed*
Experimental factors	*Not applicable*
Experimental features	*Reduced set (Reduced_ser.pdf) modified by randomly generated errors.*
Data source location	*Not applicable*
Data accessibility	*Data is contained in this article and files: Reduced_set.pdf, simulation_data.xlsx*

## Value of the data

• When there is great variability in a compiled experimental dataset, considerations on the confidence interval for the correlation coefficient r and on the maximum theoretical value achievable for r can offer hints as to what to expect from a predictive model based on that set.
• Numerical simulations used to generate a dataset of arbitrary average uncertainty and to estimate a confidence interval around the correlation coefficient r and its maximum theoretical value are easily applicable to all experimental datasets
• The here proposed data can be easily utilized to derive the range of r that can be pursued when the variability of a given dataset is known
• Along with well-known statistical parameters (such as r, r^2^, q^2^, F, SE, etc), the here proposed confidence interval of r can become a meaningful parameter to better evaluate the reliability of a given model and to understand whether there is still room for statistical improvements.

## 1 Data

Data presented here represent maximum theoretical average values and confidence interval for the correlation coefficient *r* and the determination coefficient *r*^*2*^ as obtained through numerical simulation (Table 1). The values of *r* and *r*^*2*^ correspond to different simulated levels of random error (*ε*) in the experimental data set.

**Table 1:** Maximum theoretical average values and 95% confidence interval for *r* and *r*^*2*^ from numerical simulation, at different simulated levels of random error (*ε*) in the experimental data set.

ε →	0.10	0.15	0.20	0.25	0.30
*Maximum average r*	0.97	0.94	0.90	0.86	0.81
*Confidence interval around r*	0.96–0.98	0.91–0.96	0.85–0.94	0.78–0.91	0.70–0.89
*Maximum average r*^*2*^	0.95	0.88	0.81	0.74	0.66
*Confidence interval around r*^*2*^	0.92–0.97	0.83–0.93	0.72–0.88	0.60–0.84	0.50–0.79

Original data, on which data in Table 1 are based, are contained in the files *Reduced_set.pdf* and *simulation_data.xlsx. Reduced_set.pdf* contains a set of 80 permeability coefficients k_p_ [1] assembled as the intersection of Flynn's set [2] and the Fully Validated data set [3]. The file *simulation_data.xlsx* contains data from the numerical simulation described below.

## 2 Experimental design, materials and methods

Given a set of experimental data, *y*_*i*_, we can assume that a perfect estimator ϕ for the set is known (in [1], y_i_ correspond to pk_p_ values). ϕ is a mathematical function, which correlates a set of variables {x_ij_} with the experimental value y_i_, where x_ij_ represents the j-th molecular property of the i-th molecule (Eq. (1)).

The correlation, based on a perfect estimator, yields a correlation coefficient r = 1.

(1) $$y_{i}=\phi (x_{ij})\;i=1,2\ldots n,\;j=1,2\ldots m$$

For every y_i_, we introduce an error ε·c_ik_·y_i_, where {c_ik_} is a set of normally distributed pseudo-random numbers with zero average and unitary standard deviation (obtained by applying the Box-Muller transform [4] to a set of a linearly distributed random numbers); ε corresponds to the standard deviation of the errors, normalized by y_i_.

For the k-th simulation, Eq. (1) becomes Eq. (2):

(2) $$y_{ik}=y_{i}+\varepsilon \;c_{ik}\;y_{i}=\phi_{k}(x_{ij})\;i=1,2\ldots n,\;j=1,2\ldots m,k=1,2\ldots l$$

Since ϕ_k_, by definition, is a perfect estimator, the values of r obtained for Eq. (2) in the simulation are the maximum theoretical correlation coefficients achievable given the uncertainty introduced (ε).

For different values of ε, the numerical simulation is repeated 99 times (l=99) obtaining 99 correlation coefficients r_k_ (*simulation_data.xlsx*). Table 1 shows how much r and r^2^ worsen when ε increases and confirms that the formula: maximum r^2^ ≅ (1−ε) is an approximate but yet reasonable way to estimate the worsening effect of *ε*.

As for the confidence interval around *r*, it can be estimated, for each value of *r*, by using Fisher *r*→*Z* transform [5]:

(3) $$Z=\frac{1}{2}[\;\ln(1\;+\;r)\;-\;\ln(1\;-\;r)]$$

We apply Fisher *r*→*Z* transform to the *r_k_* values, obtaining 99 *Z_k_* values. Unlike *r*, *Z* tends to a normal distribution as the number of data becomes large. Therefore, the standard deviation *S_z_* can be calculated by Eq. (4):

(4) $$S_{z}=\sqrt{\frac{1}{98}\sum_{k=1}^{99}{\left(Z_{k}\;-\;\overset{—}{Z}\right)}^{2}}$$

The 95% confidence interval around *Z* is then calculated as $\left(\overset{—}{Z}\;-\;1.96\;\cdot {\;S}_{z},\;\overset{—}{Z}\;+\;1.96\;\cdot {\;S}_{z}\right)$, and the 95% confidence interval around r is obtained from it, through the reverse transform (Eq. (5)):

(5) $$r=\frac{e^{2Z}\;-\;1}{e^{2Z}\;+\;1}$$

The confidence intervals around r and r^2^ for different values of ε are shown in Table 1.
