# Supplementary material for: Simulation data for an estimation of the maximum theoretical value and confidence interval for the correlation coefficient
Source: Data Brief. 2017 Jul 23;14:291–4. doi: 10.1016/j.dib.2017.07.045 (PMC5540710; doi:10.1016/j.dib.2017.07.045)
Supplement: Supplementary file 2 — Supplementary material [file mmc2.pdf]

The reduced data set.

The reduced data set (**Table S1**) is the intersection of Flynn's set (Flynn, 1990) and the Fully Validated set (Vecchia and Bunge, 2003). It is comprised of 80 compounds, and it is used in this work to eliminate non validated data from Flynn's set, according to the validation criteria exposed in (Vecchia and Bunge, 2003), while retaining the ability to compare results with methods optimized on Flynn's set.

**Name**	**Mass**	**logk$_P$**	**T (K)**	**t (°C)**
2,3-butanediol	90.12	-4.40	303	30
2,4,6-trichlorophenol	197.45	-1.23	298	25
2,4-dichlorophenol	163	-1.22	298	25
2-butanone	72.11	-2.35	303	30
2-chlorophenol	128.56	-1.48	298	25
2-ethoxy ethanol (Cellosolve)	90.12	-3.60	303	30
2-naphtol	144.17	-1.55	298	25
3,4-xylenol	122.16	-1.44	298	25
3-nitrophenol	139.11	-2.25	298	25
4-bromophenol	173.01	-1.44	298	25
4-chlorophenol	128.56	-1.26	298	25
4-choro-m-cresol	142.58	-1.44	298	25
4-ethylphenol	122.16	-1.46	298	25
4-nitrophenol	139.11	-2.25	298	25
aldosterone	360.44	-5.52	299	26
amobarbital	226.27	-2.64	303	30
atropine	289.37	-4.86	303	30
barbital	184.19	-3.95	303	30
benzyl alcohol	108.13	-2.22	298	25
butanoic acid	88.1	-3.00	300	27
butobarbital	212.25	-3.72	303	30
chloroxylenol	156.61	-1.23	298	25
chlorpheniramine	274.79	-2.64	303	30
cortexolone	346.46	-4.12	299	26
cortexone (deoxycorticosterone)	330.46	-3.35	299	26
corticosterone	346.46	-4.22	298	25
cortisone	360.44	-5.00	299	26
diethylcarbamazine	199.29	-3.89	303	30
ephedrine	165.23	-2.22	303	30
estradiol	272.38	-3.52	299	26
estriol	288.38	-4.40	299	26
estrone	270.37	-2.44	299	26
ethanol	46.07	-3.10	298	25
ethylether	74.12	-1.80	303	30
fentanyl	336.47	-1.94	303	30
heptanoic acid	130.18	-1.70	300	27
hexanoic acid	116.16	-1.85	300	27
hydrocortisone	362.46	-5.52	299	26

Name	Mass	$logk_p$	T (K)	t (°C)
[hydrocortisone-21-yl] hemipimelate	504.61	-2.64	310	37
[hydrocortisone-21-yl] hemisuccinate	462.53	-3.09	310	37
[hydrocortisone-21-yl] hexanoate	460.6	-1.74	310	37
[hydrocortisone-21-yl]-hydroxy-hexanoate	476.6	-3.04	310	37
[hydrocortisone-21-yl]-N,N-dimethyl-succinamate	491.64	-4.17	310	37
[hydrocortisone-21-yl]-octanoate	488.66	-1.21	310	37
[hydrocortisone-21-yl]-pimelamate	519.67	-3.05	310	37
[hydrocortisone-21-yl]-propionate	418.52	-2.47	310	37
[hydrocortisone-21-yl]-succinamate	463.59	-4.59	310	37
hydroxypregnenolone	331.2	-3.22	299	26
17α-hydroxyprogesterone	332.48	-3.22	299	26
isoquinoline	129.16	-1.77	303	30
m-cresol	108.14	-1.82	298	25
methanol	32.04	-3.30	303	30
methyl-[hydrocortisone-21-yl]-pimelate	518.64	-2.27	310	37
methyl-[hydrocortisone-21-yl]-succinate	476.56	-3.68	310	37
methyl-4-hydroxybenzoate	152.15	-2.04	298	25
n-butanol	74.12	-2.60	298	25
n-decanol	158.28	-1.10	298	25
n-heptanol	116.2	-1.46	298	25
n-hexanol	102.17	-1.89	298	25
nicotine	162.23	-1.71	303	30
n-nonanol	144.25	-1.22	298	25
n-octanol	130.23	-1.28	298	25
n-pentanol	88.15	-2.22	298	25
n-propanol	60.1	-2.85	298	25
N-nitrosodiethanolamine	134.1	-4.26	305	32
o-cresol	108.14	-1.80	298	25
octanoic acid	144.21	-1.60	300	27
ouabain	584.65	-6.11	303	30
p-cresol	108.14	-1.75	298	25
pentanoic acid	102.13	-2.70	300	27
phenobarbital	232.24	-3.35	303	30
phenol	94.11	-2.09	298	25
pregnenolone	316.48	-2.82	299	26
progesterone	314.46	-2.82	299	26
resorcinol	110.11	-3.62	298	25
salicylic acid	138.12	-1.89	303	30
scopolamine	303.53	-4.20	303	30
sufentanyl	386.55	-1.92	310	37
testosterone	288.42	-3.40	299	26
thymol	150.22	-1.28	298	25

Table S1. The reduced data set. $logk_p$ is the logarithm of the permeation coefficient k_p, where k_p is expressed in cm/h. T and t are the experimental temperatures in K and °C, respectively.

References

Flynn, G.L., 1990. Physicochemical determinants of skin absorption, in: Garrity, T.R., Henry, C.J. (Eds.), Principles of Route to Route Extrapolation for Risk Assessment. Elsevier, New York, pp. 93–127.

Vecchia, B.E., Bunge, A.L., 2003. Evaluating the Transdermal Permeability of Chemicals, in: Guy RH, H.J. (Ed.), Transdermal Drug Delivery. CRC Press, New York, pp. 38–39.
